# Using eLearning to improve and retain the knowledge of community health workers in maternal and neonatal health in Rwanda: A cohort study

**DOI:** 10.1002/puh2.174

**Published:** 2024-04-28

**Authors:** Yves Sangwa, Victor Ndaruhutse, Samson Radeny, Dieudonne Ndatimana, François Niragire, Erigene Rutayisire, Beatrice Mukamana, Josee Uwamariya, Clovis Kabanda, Angelique Nyirafaranga, Marie Chantal Uzayisenga, Chris Adrien Kanakuze, Jean Claude Gasamagera, Jacqueline Umunyana, Christian Mazimpaka

**Affiliations:** ^1^ IntraHealth International Kigali Rwanda; ^2^ Department of Applied Statistics University of Rwanda Kigali Rwanda; ^3^ Department of Community Health University of Rwanda Kigali Rwanda; ^4^ Rwanda Biomedical Center Kigali Rwanda; ^5^ Department of Midwifery University of Rwanda Kigali Rwanda

**Keywords:** community health workers, community‐based maternal and neonatal health, eLearning, Rwanda, smartphones, training

## Abstract

**Background:**

In Rwanda, community health workers (CHWs) serve a crucial function in providing community‐based maternal and neonatal health (CBMNH) services. However, limited access to continuous training affects their confidence and ability to execute their roles effectively. This study aimed at evaluating the impact of eLearning on enhancing and maintaining CHWs’ knowledge of CBMNH.

**Methods:**

This cohort study, conducted from April to October 2021 in two Rwandan districts, evaluated knowledge acquisition and retention among 36 CHWs participating in an eLearning course. Knowledge scores were measured using a structured questionnaire administered pre‐training, post‐training and at a 6‐month follow‐up. Descriptive analysis and paired *t*‐tests were used to assess mean score differences.

**Results:**

There was improvement in CHWs’ performance scores following eLearning training, with an average increase from 86.5% to 98.2%. The improvement was sustained at a 6‐month follow‐up. Statistical significance was found between age category and CHWs’ pre‐ and post‐test performance (*p* = 0.01, *p* = 0.04 respectively), and between years of experience and pre‐test scores (*p* = 0.02).

**Conclusions:**

The results of this study suggest that eLearning is an effective method for enhancing and retaining CHWs’ knowledge of CBMNH. The findings support the use of eLearning as a valuable strategy for strengthening the capacity of CHWs in Rwanda and other countries with similar contexts.

## Graphical abstract

The study showed a significant increase in average scores from 86.48% in the pre‐test to 98.20% post‐test and 97.75% at follow‐up, indicating effective knowledge acquisition and retention among ASMs. The mean score differences were 11.72 and 11.27, respectively (*p* < 0.001), with no significant changes between post‐test and follow‐up (−0.46; *p* = 0.376).

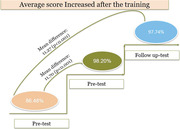

## INTRODUCTION

Community health workers (CHWs) have garnered global recognition for their role in delivering primary healthcare services in regions where healthcare resources and personnel are insufficient. This is predominantly the case in low‐ and middle‐income countries (LMICs) where healthcare systems face an array of challenges, including a lack of sufficient human resources for health [1]. In these settings, CHWs play an important role especially in improving access to maternal and neonatal healthcare services [2, 3].

However, the lack of continuous training opportunities affects the quality of services that they can provide [2, 3]. Traditional classroom training has been a long‐standing method of instruction for CHWs, and it offers numerous benefits, such as face‐to‐face interaction and immediate feedback. However, in addition to its high cost and associated logistical challenge, it does not effectively ensure the retention of knowledge and skills over time [4]. This logistical challenge not only places an additional burden on CHWs but also inadvertently deprives communities of access to primary healthcare services during the training period [5]. Additionally, refresher trainings to update and reinforce knowledge and skills, acquired during these trainings, are often infrequent and heavily dependent on the availability of funds [5].

In view of the increasing availability of technology infrastructure globally, the WHO recommends eLearning as an alternative to increase training access to healthcare workers [6]. Studies suggest that eLearning using mobile phones can effectively contribute to training healthcare workers [7, 8, 9, 10, 11]. eLearning is user‐friendly, flexible and enhances access to standardized content [12, 13]. It allows for learning at one's own pace and provides the flexibility to review complex concepts multiple times, allowing better understanding and retention of knowledge [12, 13].

In Rwanda, CHWs are an integral part of the healthcare system. Each village, the smallest administrative unit in the country, is served by a team of four CHWs [14]. This team includes two CHWs called binome—a male–female pair responsible for offering a range of general health services to the community, an animatrice de Santé Maternelle (ASM) who provides maternal and newborn health services, and a fourth CHW focuses on delivering health promotion services, educating the community about various health issues and preventive measures [14]. Upon recruitment, CHWs in Rwanda undergo initial didactic classroom training at local health centres and must successfully pass a post‐training assessment by achieving a score of 80% or higher. The training for Binomes focuses on Integrated Community Case Management (iCCM) and Community–Based Provision of Family Planning Services (FP), whereas the training for ASMs focuses on community‐based maternal and newborn health (CBMNH) [14].

Despite the comprehensive nature of the initial training, the provision of regular refresher training constitutes a considerable challenge, primarily due to logistical and financial constraints. These challenges are exacerbated by the extensive distribution of more than 50,000 CHWs across the country and a high turnover rate, necessitating frequent training sessions [15, 16]. To contribute to addressing this challenge, the USAID Ingobyi Activity, led by IntraHealth International, collaborated with the Ministry of Health (MOH) and the Rwanda Biomedical Centre (RBC) to pilot an eLearning training approach using smartphones in two districts. The primary objective was to enhance the quality and frequency of refresher trainings, providing a flexible and accessible means of continuous education to support the current didactic refresher trainings. The aim of this study is to assess the effectiveness of this eLearning approach on the knowledge retention and performance of CHWs in their delivery of CBMNH services.

## METHODS

### Study setting

The study was piloted in two Rwandan districts: Rutsiro, located in the Western province, represents a rural setting, whereas Ngoma, in the Eastern Province, represents a Peri‐Urban district. Together, these two districts comprise 958 villages [17], each served by one CHW (ASM) who delivers CBMNH services to an average of 100 households [14]. In terms of infrastructure, the districts of Ngoma and Rutsiro have electricity coverage rates of 58.3% and 50.2%, respectively [18].

### Description of the intervention

In collaboration with RBC, we used an existing MOH eLearning platform—an open‐source learning management system based on the Moodle platform. Existing community health training manuals were reviewed and tailored to meet the knowledge and skills needed by CHWs. From the training manuals approved by the MOH, USAID Ingobyi Activity's eLearning team re‐configured the training materials and developed the audio–visual content that is easy to navigate, interactive and accessible to all CHWs, including those with limited digital literacy level. The process involved scripts writing, field photo shooting followed by recording scripts into audio formats and videos, and studio editing to add appropriate images to the audio and visual text. After editing and adapting the content, the final version of the course was uploaded to the eLearning platform.

The course materials were reviewed in a series of stakeholder workshops and validated by the community health technical working group before implementation. Inputs from CHWs and national trainers were captured into the eLearning content. Subsequently, an offline version of the platform was generated to facilitate access to the training content when the internet connectivity is slow or unavailable. Prior to training, USAID Ingobyi Activity procured and distributed locally made smartphones, Mara Z and Mara Z1, to the selected CHWs to ensure that learners had easy access to training content. Thereafter, the eLearning team conducted a digital literacy assessment to gauge learners’ digital knowledge and conducted orientations to ensure that CHWs were well versed with their smartphones and the eLearning content.

Following the preparatory phase, USAID Ingobyi Activity collaborated with MOH to pilot the eLearning CBMNH courses in six health centres (Rukira, Nyange and Jarama in Ngoma district, and Kinihira, Kayove and Congo Nil in Rutsiro district). Purposively selected 36 CHWs in charge of maternal health (ASMs) undertook the course. CHWs were required to complete the course within 4 weeks via the MOH eLearning platform, utilizing a self‐paced methodology. CHWs took a pre‐test before accessing the course, after completing the course and at 6 months completing the course.

### Content of the eLearning course

Using eLearning approach, CHWs underwent training on CBMNH course which included four distinct learning units:

1. **Overview on maternal and newborn health**: This unit focused on the causes of maternal and newborn deaths and provided a comprehensive understanding of maternal and newborn health. The unit further focused on ASMs’ roles, responsibilities and tools used during pre‐ and post‐natal care (PNC) home visits.
2. **Conducting survey of girls and women in childbearing age**: The unit emphasized guideline‐compliant preparation for door‐to‐door visits, proper register documentation, education on FP and sexual reproductive health for women and girls of childbearing age, proper usage of urinary pregnancy tests and protocol‐based counselling and educating pregnant women on antenatal care and facility‐based delivery.
3. **Conducting home visits for pregnant women**: This unit focused on comprehensive follow‐up care for pregnant women, including consistent monitoring, guideline‐based follow‐up, danger sign identification, nutrition, care provision and timely referrals.
4. **Conducting post‐natal care visit**: The unit focused on preparing mothers and families for newborn care post‐delivery, conducting home visits to mothers and newborns as per PNC protocols, including an early detection of disabilities and special needs in newborns, sensitizing mothers on positive parenting, early child stimulation, exclusive breastfeeding and breastfeeding practices.

### Study design and population

This study employed a prospective cohort design to evaluate the efficacy of eLearning programmes in enhancing the knowledge of female CHWs engaged in CBMNH in the Ngoma and Rutsiro districts. Conducted between April and October 2021, the study was piloted on 36 active CHWs. These participants were purposively selected on the basis of their completion of basic CBMNH training at least 1 year before the study and the absence of any refresher training in the year leading up to the study.

### Data collection and analysis

Demographic data were collected using the questionnaire and entered into a Microsoft Excel dataset prior to the start of eLearning training. Data related to individual performance scores in the pre‐ and post‐ and follow‐up tests were collected using Moodle in the eLearning platform and were downloaded to Excel. The data from Moodle were combined with the dataset that included participants’ demographic and test results information. Data were analysed using Stata version 17. Descriptive analysis was conducted, and data were presented using frequency and percentage to describe the characteristics of participants. The mean scores across all test scores were computed. A series of paired sample *t*‐tests was conducted to measure the mean difference across the three tests’ results. A confidence interval of 95% was used, and *p*‐value of 0.05 was estimated and used to measure the statistical significance of the mean difference. We assessed the relationship between each participant's characteristics and changes in test scores for pre‐, post‐ and follow up tests using cross tabulation.

## RESULTS

A total of 36 ASMs, 18 from each of the two districts, participated in this study. All of them were female and had completed at least the primary education level. A total of 16 (44.4%) participants were between the ages of 35 and 44 years, with the average age being 42.7 years old (SD ± 8.5). The majority (63.9%) had more than 10 years in their role. Most of the participants had electricity in their homes to be able to charge their smartphones (55.6%). Others charged their phones at the house of one of their neighbours (81.2%). Finally, all participants reported to have never used a smartphone before the study (Table 1).

**TABLE 1:** Characteristics of community health workers (CHWs) interviewed to evaluate the use of eLearning to improve and retain their knowledge in maternal and neonatal health in Rwanda, April–October 2021 (*n* = 36).

Variable	Frequency	%
**District of work**		
Rutsiro	18	50.0
Ngoma	18	50.0
**Gender**		
Female	36	100.0
Male	0	0.0
**Level of education**		
Primary	36	100.0
Ordinary (O) level secondary	0	0.0
**Age category (years)**		
25–34	5	13.9
35–44	16	44.4
45–54	12	33.3
≥55	3	8.3
**Years of experience as CHW (years)**		
≤10	13	36.1
>10	23	63.9
**Electricity at home**		
Yes	20	55.6
No	16	44.4
**Place of charging smartphone if no electricity at home**		
Neighbour	13	81.2
In a commercial centre	3	18.8
At health centre	0	0.0
**Previous experience with smartphone use**		
Used before	0	0.0
Never used it	36	100.0

Abbreviation: O'level, ordinary level.

Following the eLearning intervention, CHWs demonstrated substantial improvements in their knowledge, with post‐intervention and 6‐month follow‐up tests showing an increase in correct answers across most evaluated topics. Correct answers increased from below 65% pre‐intervention to over 97% in both post‐intervention and 6‐month follow‐up tests in key domains of neonatal and maternal health, antenatal care and essential newborn care (Table 2).

**TABLE 2:** Community health workers (n = 36) who provided correct answers to each of the evaluation questions at the key time points of the study period to evaluate the use of eLearning to improve and retain knowledge of community health workers (CHWs) in maternal and neonatal health in Rwanda, April–October 2021.

No	Question	Pre‐test (counts (%))	Post‐test (count (%))	Follow‐up test (count (%))
1	Among the following, what are the causes of neonatal deaths? Select all that apply	21 (58)	35 (97)	36 (100)
2	What are Causes of maternal deaths? Select all that apply	18 (50)	35 (97)	36 (100)
3	As recommended by WHO, how many times a pregnant woman should consult for ANC during her pregnancy period?	21 (58)	35 (97)	35 (97)
4	Select the most important services that a pregnant woman receives from a nurse when she goes for ANC at health facility?	20 (56)	36 (100)	35 (97)
5	Select three possible danger signs for a pregnant woman	36 (100)	35 (97)	35 (97)
6	Select key actions/services in caring for a young pregnant girl (teen)?	36 (100)	35 (97)	36 (100)
7	From the following, select the activities that a CHW can do to encourage women to delivery at health facility	21 (58)	36 (100)	36 (100)
8	Select three danger signs for the newborn after birth	23 (64)	34 (94)	36 (100)
9	When is a baby born underweight?	21 (58)	36 (100)	36 (100)
10	Which of the following are the most important steps/services a CHW should take to support a low‐birth‐weight baby?	23 (64)	35 (97)	35 (97)
11	What are the most important measures to protect the baby from the cold after birth?	21 (58)	36 (100)	36 (100)
12	If a 3‐day‐old baby likes to sleep, when you try to wake up her/him you realize that she/he is weak, what should be the problem?	9 (25)	35 (97)	34 (94)
13	What can a maternal CHW do for that baby?	36 (100)	23 (64)	26 (72)
14	What can a maternal CHW do for a mother who complains of breast pain?	19 (53)	35 (97)	36 (100)
15	What can the maternal CHW do to keep the baby warm?	6 (17)	36 (100)	36 (100)
16	Select the simple signs for a pregnant woman or mother with mental problems	19 (53)	34 (94)	32 (89)
17	Select the most important signs for a pregnant woman or mother with mental disorder	22 (61)	35 (97)	36 (100)
18	Select the reasons why CHW's home visits to women after delivery are so important	20 (56)	32 (89)	33 (92)
19	Select the danger signs that may appear in a woman/girl before an abortion	21 (58)	33 (92)	36 (100)
20	Select three danger signs that may appear in a woman/girl after abortion	22 (61)	36 (100)	35 (97)
21	When is a CHW allowed to perform a urine pregnancy test (UPT) to determine whether a woman/girl is pregnant?	18 (50)	33 (92)	31 (86)
22	Select the main equipment/tools needed for a CHW to perform a urine pregnancy test (UPT)	23 (64)	35 (97)	36 (100)
23	Select the steps followed by a CHW when performing a urine pregnancy test	6 (17)	32 (89)	27 (75)
24	A greater number of maternal deaths are caused by post‐partum haemorrhage compared other causes of maternal mortality. (Yes, No)	36 (100)	35 (97)	33 (92)
25	It is easy to know that a mother will have PPH after giving birth. (Yes, No)	36 (100)	36 (100)	36 (100)
26	Postpartum haemorrhage is a problem when a mother changes her underwear 2 times in less than an hour. (Yes, No)	22 (61)	36 (100)	36 (100)
27	A mother can take misoprostol after giving birth to one child without checking that there remains another one. (Yes, No)	36 (100)	36 (100)	35 (97)
28	A mother can take misoprostol before the delivery of the placenta (Yes, No)	22 (61)	35 (97)	34 (94)
29	A mother can take misoprostol within 3 h after giving birth. (Yes, No)	23 (64)	36 (100)	35 (97)
30	Misoprostol can prevent post‐partum haemorrhage (Yes, No)	36 (100)	36 (100)	35 (97)
31	The best time to give misoprostol to a mother is when she is seven months pregnant (Yes, No)	36 (100)	36 (100)	36 (100)
32	The role of misoprostol is to induce uterine contractions (Yes, No)	22 (61)	36 (100)	36 (100)
33	If the mother has taken misoprostol, it is not necessary to go to the health facility (Yes, No)	21 (58)	35 (97)	33 (92)

The average score increased from 86.48% in the pre‐test to 98.20% in the post‐test with a mean difference of 11.72 (*p *< 0.001). Moreover, participating ASMs significantly retained their knowledge over the 6‐month period following the completion of the course. The average score increased from 86.48% in the pre‐test to 97.75% in the follow‐up test with a mean difference of 11.27 (*p *< 0.001). However, there was no significant knowledge improvement between the post‐ and follow‐up test (−0.46; *p *= 0.376) (Table 3).

**TABLE 3:** Comparison of mean differences in pre‐, post‐ and follow‐up test to evaluate the use of eLearning to improve and retain knowledge of community health workers (CHWs) in maternal and neonatal health in Rwanda, April–October 2021 (n = 36).

	Mean, SD	95% CI		*p* Value	Cohen's *d*
Pre, mean (SD)	86.48 (4.2)	85.04	87.92		
Post, mean (SD)	98.20 (2.0)	97.52	98.88		
Follow‐up, mean (SD)	97.75 (2.7)	96.81	98.70		
Pre–post difference, mean (SD)	11.72 (4.5)	10.18	13.25	**≤0.001**	3.52
Post–follow‐up difference, mean (SD)	0.44 (2.9)	0.56	1.45	0.376	0.18
Pre–follow‐up difference, mean (SD)	11.27 (5.4)	9.44	13.10	**≤0.001**	3.13

*Note*: Bold value indicates the statistical significance of *p* < 0.05.

Abbreviations: CI, confidence interval; SD: standard deviation.

The study found a significant association between age groups and test scores, both in pre‐test (*p* = 0.01) and follow‐up test results (*p* = 0.04). In the pre‐test, the younger age group (25–34 years) outperformed the older group (55+ years) with average scores of 89.5%–81.8%, respectively. However, in the follow‐up test, this trend reversed, with the older age group scoring higher (99.0%) than the younger (95.4%). Both groups improved from pre‐test to follow‐up, but the older age group demonstrated a more consistent improvement across all tests (pre‐test: 81.8%, post‐test: 96.8%, follow‐up: 99.0%), unlike the younger group, which saw a slight decline from post‐test (98.5%) to follow‐up (95.4%). There was a significant association between years of experience and pre‐test scores, with ASMs less than 10 years of experience scoring higher (88.5%) than their counterparts with more than 10 years (85.2%) in the pre‐test (*p* = 0.02). No significant differences were observed in post‐test (*p* = 0.58) and follow‐up tests (*p* = 0.39) scores across experience levels. The study did not find a significant association between test performance and availability of electricity at a CHW's home (Table 4).

**TABLE 4:** Association between community health workers’ (CHWs’) characteristics and test results in the study to evaluate the use of eLearning to improve and retain knowledge of CHWs in maternal and neonatal health in Rwanda, April–October 2021 (n = 36).

Variable	Pre‐test		Post‐test		Follow‐up test	
	Mean (SD)	*p* Value	Mean (SD)	*p* Value	Mean (SD)	*p* Value
**Age category (years)**						
25–34	89.5 (1.0)	**0.01**	98.5 (1.4)	0.96	95.4 (4.8)	**0.04**
35–44	86.7 (3.7)		97.7 (2.2)		97.7 (2.6)	
45–54	85.9 (5.0)		98.9 (1.3)		98.4 (1.9)	
>=55	81.8 (3.4)		96.8 (3.3)		99.0 (0.8)	
**Experience as CHW** (years)						
<=10	88.5 (2.9)	**0.02**	97.9 (2.2)	0.58	97.2 (3.3)	0.39
>10	85.2 (4.5)		98.3 (1.8)		98.0 (2.4)	
**Electricity at home**						
No	85.4 (4.7)	0.20	97.7 (1.9)	0.25	97.2 (2.5)	0.28
Yes	87.3 (3.7)		98.5 (2.0)		98.2 (2.9)	

*Note*: Bold value indicates the statistical significance of *p* < 0.05.

Abbreviations: *p* Value, probability value; SD, standard deviation.

## DISCUSSION

The findings of this study echo those of prior research, particularly regarding the efficacy of eLearning in improving and maintaining knowledge across various demographics [19]. The fact that ASMs, despite having no previous smartphone experience, significantly improved their knowledge following the eLearning course is testament to the flexibility and robustness of eLearning as an instructional method.

The influence of age on learning outcomes is an intriguing facet of this study and provides an opportunity to contrast with findings from other research. Older adults often encounter difficulties with technology‐based learning [20], an observation that could seem at odds with the results of this study, in which the older ASM group not only significantly improved their performance, but also exceeded the younger group in the follow‐up test. However, this deviation may potentially be attributed to the distractions that younger individuals may face, particularly with the omnipresence of social media [21]. This hypothesis suggests that younger individuals, while technologically adept, may be prone to diversion from educational content, impacting the retention of knowledge in long term.

This study observed a correlation between years of experience and learning outcomes. A study on effectiveness of web‐based virtual learning found that mature professionals often have more structured knowledge frameworks, possibly inhibiting new learning [22]. This pattern mirrors our observations with the more experienced ASMs exhibiting a lower pre‐test performance. However, the uniform increase in scores across both experience groups in the post‐ and follow‐up tests underscores the effectiveness of eLearning for all learners, irrespective of their professional experience.

The result of this study demonstrates the efficacy of smartphone‐based eLearning in enhancing the knowledge of CHWs, particularly in CBMNH competencies. As the Rwandan government pursues the integration of eLearning at the community level, these findings substantiate this approach as a cost‐effective, sustainable strategy for improving community health programmes. With the upward trajectory of smartphone utilization in LMICs, these devices offer a promising avenue for enhancing CHW training and sustaining knowledge retention.

Limitations for this study include small sample size and the inclusion of 2 out of 30 districts, which may limit the generalizability of the results. Nevertheless, it offers significant insights into the domain of technology‐based learning, particularly through the novel examination of knowledge acquisition and retention among CHWs using smartphones. To our knowledge, this is the first study conducted to assess the knowledge acquisition and retention of CHWs using smartphones. The nuanced insights provided by this study, especially about the impacts of age, experience and resource availability on eLearning outcomes, are of significant value for educators, policymakers and the wider field of technology‐based learning. They challenge preconceived notions and shed light on new areas for research.

## AUTHOR CONTRIBUTIONS

Yves Sangwa, Victor Ndaruhutse, Christian Mazimpaka, Jacqueline Umunyana, Samson Radeny, and Dieudonne Ndatimana conceived the study. Beatrice Mukamana, Clovis Kabanda, Angelique Nyirafaranga and Marie Chantal Uzayisenga contributed in the data collection. Yves Sangwa, Victor Ndaruhutse, Christian Mazimpaka, Jacqueline Umunyana, François Niragire, Erigene Rutayisire, Josee Uwamariya, Dieudonne Ndatimana and Beatrice Mukamana undertook a review of literature and data analysis. All authors supported data interpretation and manuscript writing. All authors reviewed and approved the final manuscript.

## CONFLICT OF INTEREST STATEMENT

Authors declare that they have no conflicts of interest.

## ETHICS STATEMENT

All study participants provided signed informed consent in the local language (Kinyarwanda) prior to data collection. We received ethical approval from the Rwanda National Ethics Committee (Kigali, Rwanda, No: 48/RNEC/2022) as well as from IntraHealth International's Institutional Review Board (Chapel Hill, North Carolina, 27517, United States, No: 21006).

## Data Availability

The datasets for the current study are available from the corresponding author on request.
